# “French Phage Network” Annual Conference 2018—Fourth Meeting Report

**DOI:** 10.3390/v11050470

**Published:** 2019-05-23

**Authors:** Rémy Froissart, Charlotte Brives, Cécile Breyton, Claire Le Marrec

**Affiliations:** 1 Centre national de la recherche scientifique (CNRS), Institut de recherche pour le développement (IRD), University of Montpellier, UMR 5290, MIVEGEC, F-34394 Montpellier, France; 2 Centre national de la recherche scientifique (CNRS), Centre Emile Durkheim—UMR5116, 33607 Bordeaux, France; charlottebrives@gmail.com; 3 Institute for Structural Biology (IBS), Commissariat à l’énergie atomique (CEA), Centre national de la recherche scientifique (CNRS), University of Grenoble Alpes, 38000 Grenoble, France; Cecile.Breyton@ibs.fr; 4 Institute of Vine and Wine Science (ISVV), EA 4577 Œnologie, University of Bordeaux, 33882 Bordeaux INP, France; Claire.LeHenaff@enscbp.fr; 5 Institut National de la Recherche Agronomique (INRA), USC 1366, 33146 Villenave D’Ornon, France

**Keywords:** bacteriophage, bacteria, infection, co-evolution, virulence, resistance, bacteriophage therapy, structural biology, genomics, France

## Abstract

The present meeting report aims to cover the scientific activities of the 4th French Bacteriophage Network (Phages.fr) symposium which took place during 24th–25th September 2018, at the Agora du Haut-Carré in Talence (France). The hosting institute was University Bordeaux and 72 participants attended the meeting from both public and private sectors, coming from France, Belgium, Ireland, Germany, Portugal and Canada. The scientific program was structured in three themed oral sessions entitled “ecology and evolution”, “bacteriophage-host molecular interaction”, and “therapy and biotechnology applications” consisting of 21 oral presentations, including three keynote lectures, and a presentation of the activities of the Spanish bacteriophage network. A poster session included 22 presentations.

## 1. Introduction

As we all know but tend to forget, bacteriophages were involved in several central discoveries in biology during the last century. Indeed, study of bacteriophages led to the discovery that mutations are at the basis of genetic variations [1], that DNA is the basis for genetic transmission [2], as well as the discovery of restriction enzymes as a matter of bacterial resistance to their bacteriophage predators [3]. The latest breakthrough involving bacteriophages came from the bacterial resistance mechanisms involving the clustered regularly interspaced short palindromic repeats (CRISPR) [4]. Interestingly enough, these discoveries and many others are now widely used in biology (evolutionary biology) and more particularly in molecular biology and biotechnology. Moreover, the current context of a worldwide increase of therapeutic failures leading to human deaths or severe disabilities (such as amputations) due to pathogenic bacteria resistant to all available antibiotics, has triggered a renewed clinical and agricultural interest in bacteriophages, as therapeutic complements and/or alternatives [5] against bacterial infections. This reinforces the need to create opportunities for dialogue and exchanges of experience between actors, and also to discuss the need for an adapted (or entirely new) regulatory framework for the reintroduction of bacteriophage therapy, in Europe, and other countries.

As an introduction, Claire Le Marrec summarized the different activities of the French network in 2018, recalling the two workshops organized during 2018 dedicated to the ecology of bacteriophages (Nancy) and electron microscopy for bacteriophage study (Grenoble). Also, specialists in computational biology organized a “viromathon” consisting of the analysis of same raw metagenomic data by different groups (both long reads with Pac-Bio and short reads with Illumina) leading to virome assembly and annotation. Data will be presented in the next meeting in 2019 (October 8–9 in Grenoble). Last, the projects of two young colleagues of the network were selected and funded by the French National Research Agency (ANR) in 2018: “Anthropophage”, a multi-site ethnography of the current development of applications of bacteriophages in human, animal and plant health (Charlotte Brives, CNRS, UMR 5116 Bordeaux) and “BacPhageChat”, the deciphering of the crosstalk between bacteria and prophages (Aurélia Battesti, Laboratoire de Chimie Bactérienne, UMR 7283, Marseille).

**Figure 1:**
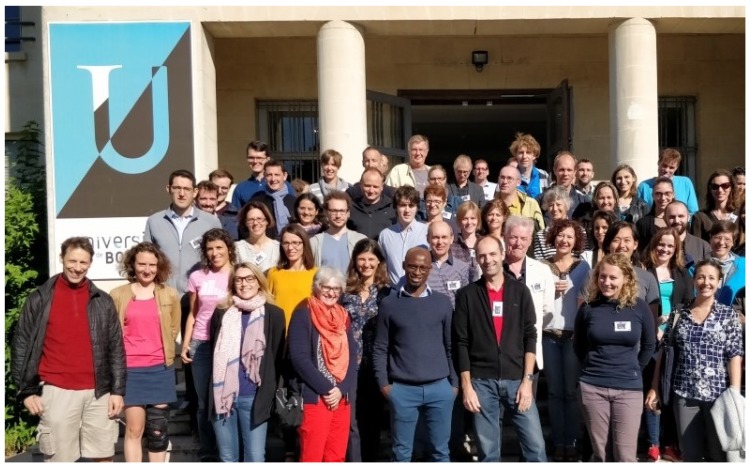
Overview of the participants of the 4th French bacteriophage Network (bacteriophages.fr) at the University Bordeaux in 2018.

The scientific program of our annual meeting was structured in three themed oral sessions entitled “ecology and evolution”, “bacteriophage-host molecular interaction”, and “therapy and biotechnology applications”. We recalled hereafter the meanings of some of the 21 oral presentations, including three keynote lectures. A poster session included 22 presentations (Table 1). Afterall, 72 participants attended the meeting from both public and private sectors, coming from France, Belgium, Ireland, Germany, Portugal and Canada (Figure 1). 

## 2. Ecology and Evolution

Following the opening address of the symposium, **Patrick Forterre** (Institut Pasteur, Paris) opened the first session with his key note lecture entitled “Drawing viral “trees of life” that raise intriguing evolutionary questions”. His presentation brought forward a number of questions regarding the definition of a virus (a capsid encoding organism?), its functional state (the virocell) and how it is possible to place viruses in universal trees of life [6]. P. Forterre presented preliminary attempts to decipher, through phylogenetic analyses, the history of two major lineages of DNA viruses present in the three cellular domains, Archaea, Bacteria and Eukarya: the Adenovirus/PRD1 and the HK97 lineages, both defined by their major capsid proteins and packaging ATPases. Forterre and colleagues obtained universal trees of life of the PRD1 and HK97 «super lineages» that are at odds with either the classical Woese’s tree of cellular domains or the fashionable (but probably wrong) 2D (eocyte) tree. Their results raise exciting questions about the role of viruses in the evolution of the three cellular domains, and, as some lineages seem to have co-evolved with proto-eukaryotes, their role in the formation of modern eukaryotes.

Three presentations were next dedicated to the impact of prophages on the physiology of the bacterial host. **Nicolas Ginet** (CNRS, Laboratoire de Chimie Bactérienne UMR 7283, Marseille) presented results regarding horizontal gene transfers (HGT) that enable acquisition and evolution of new abilities coming from other—sometimes distantly phylogenetically related—organisms. Working on the model magnetotactic bacteria *Magnetospirillum magneticum* AMB-1 (*i.e.*, bacteria able to navigate in the chemically stratified aquatic environments along the geomagnetic field lines), N. Ginet presented evidence that three putative prophages were present in the genome of this bacterium. One of these prophages (named FRODO) can spontaneously excise from its host genome at low frequencies and form viral particles. Interestingly, this prophage transports magnetotaxis-related genes clustered in a Magnetotactic Islet specifically belonging to this strain, suggesting that some magnetotactic traits can be horizontally transferred and acquired thanks to temperate bacteriophages.

**Adélaïde Renard** (graduate student in the group of Nathalie Van Der Mee-Marquet, UMR 1282, Tours) assessed the complex effect of a prophage harbored by pathogenic members of the *Streptococcus agalactiae* group B (GBS). She focused on the ability of such lysogens to outcompete commensal bacteria such as *Lactobacillus crispatus* in healthy vagina. GBS (associated with the clonal complex CC17) has become, since the 1960s, the leading cause of neonatal infection in industrialized countries. Using whole genome sequencing of 14 GBS strains representative of the species, Adélaïde Renard and colleagues identified 22 prophages clustered into 6 groups, A to F. Interestingly, CC17 GBS strains frequently were observed to carry A-prophages and their integration sites were located near bacterial genes involved in adaptation, stress resistance or virulence. In addition, prophages were shown to carry genes specifying proteins involved in defense systems, adaptive response and virulence. By comparing two isogenic strains that differ only by the lysogenic presence of an A type prophage, the authors observed that the lysogenic strain exhibited (1) a higher maximal growth rate, (2) a shorter generation time when cultivated in the presence of *L. crispatus* supernatant, and (3) a higher ability to produce biofilm. Further investigations are currently being performed to investigate the molecular mechanisms associated with the observed phenotypes.

**Astrid Wahl** (post-doc in the group headed by Mireille Ansaldi, Laboratoire de Chimie Bactérienne UMR 7283, Marseille) aims to complete our knowledge of the general strategies responsible for lysogeny maintenance of prophages in bacterial genomes. Astrid Wahl and colleagues investigated the effect of global regulators on the maintenance of *Salmonella enterica* serovar *typhimurium* specific prophages. *S. enterica* genomes usually carry 4–5 functional prophages and their genes make up around 30% of the pool of accessory genes. The authors quantified the excision level of functional and defective prophages in various mutants of these bacterial regulators. They thus observed that HNS (a nucleoid associated protein), a well-known negative regulator of HGT, negatively controls the excision of the Gifsy1 prophage. HNS was subsequently shown to target the Gifsy1-encoded anti-repressor protein coded by gfoA, a protein produced when the LexA-dependant SOS-response is activated. A. Wahl thus suggested a model where in the absence of HNS, more GfoA anti-repressor protein is produced, which in turn sequesters the GfoR repressor leading to excision of Gisfy1. Further studies will aim to clarify if HNS directly regulates gfoA and if this is the case, where exactly does HNS bind in comparison to LexA, for whom a well conserved binding box is present in the promoter region of gfoA. 

**Marianne De Paepe** (INRA, Institut MICALIS UMR1319, Jouy-en-Josas) evaluates the impact of temperate bacteriophages on the bacterial population dynamics in the gut of gnotoxenic mice. M. De Paepe presented elegant experiments where they monitored the population dynamics of a dominant gut microbiota species, *Roseburia intestinalis* L1–82, and its bacteriophage Shimadzu. Shimadzu is initially a *R. intestinalis* resident prophage. It was shown to systematically acquire mutations in the lysis/lysogeny regulatory region and evolve towards ultravirulence (the ability to infect a lysogen) in a simplified gut ecosystem. Ultravirulent bacteriophages were also shown to acquire mutations in their tail fiber, thanks to a diversity-generating retroelement (DGR). In mice, bacteriophage infection killed the majority of susceptible bacteria in a few days, leading to the natural selection of bacteriophage resistant bacteria. Resistance was due to the rapid acquisition of a new spacer in their CRISPR loci (observed in several independent lineages). These results indicated that bacteriophages are important killers of the gut symbiont *R. intestinalis* in vivo with a fast arm-race between the two protagonists. As the studies were conducted in a minimal community, the perspectives are to assess the impact of bacteriophages on *R. intestinalis* densities and diversity in “real life”.

**Sylvain Gandon** (CNRS, Centre d’Écologie Fonctionnelle et d’Évolution UMR 5175, Montpellier) presented results from his group focusing on in vitro experimental evolution. Their objective was to raise fundamental issues as to how the diversity of pathogens, as well as hosts (in terms of their resistance to the pathogens), can have significant effects on epidemics [7]. To address this, an experimental system was set to monitor the influence of bacteriophage exposure on bacterial population dynamics and CRISPR resistance diversity. More specifically, scientists produced a polymorphic bacterial population composed of bacterial genotypes harboring different spacers in their CRISPR locus (each bacterial genotype being resistant to a bacteriophage harboring the corresponding sequence of the spacer). Its sensitivity was assessed towards different bacteriophages, targeted by the CRISPR system. In the absence of bacteriophages, the diversity of resistance was very quickly lost in the polymorphic bacterial populations. This was due to the natural selection among bacterial genotypes. The exposure of bacteria to an undiversified bacteriophage population also led to a loss of diversity in the short term. However, diversity was restored after a few days due to the acquisition of new resistance in some bacterial genotypes. Finally, exposure to a diverse bacteriophage population maintained the initial bacterial diversity of resistance and quickly lead to significant differentiation between host populations. These results illustrate the impact of the selection pressure imposed by bacteriophages on the population dynamics and evolution of their hosts. They also demonstrate that antagonistic co-evolution can play a major role in maintaining local diversity and differentiating between populations.

The association of bacteriophages and their cognate hosts from genomic data of natural communities is still a challenge for computational analysis. **Stéphane CHAILLOU** (INRA, Institut MICALIS UMR1319, Jouy-en-Josas) aimed to characterise the spatio-temporal variations across bacteriophages and bacterial populations in a complex food ecosystem. Stéphane Chaillou and his colleagues sampled a cheese surface (rind of smear-type cheese “Epoisses”) under different temperatures and concentrations of nitrate and oxygen. DNA was extracted and sequenced using a shotgun pair-end MiSeq sequencing (for bacteriophage genome assembly) as well as bacterial internal transcribed spacer and 16S rDNA V3-V4 amplicon sequencing. They observed the presence of well-known bacteriophages infecting *Lactococcus lactis* (such as members of the 943 group, with a frequency of 28% of the reads) as well as unknown bacteriophages (64% of reads). Interestingly, through the careful examination of the frequencies of both bacteriophages and bacteria in each condition, new assignments for bacteriophage-host relationships could be proposed (even with unknown bacteriophages).

**Marie-Agnès Petit** (INRA, Institut MICALIS UMR1319, Jouy-en-Josas) reported on the influence of bacteriophages on the early life microbiota colonization and maturation, and assessed whether bacteriophages may play a role in the risk of later asthmatic disease development in human. The authors focused on *Escherichia coli* bacteriophages, as *E. coli* is one of the first bacterial species to colonize the infant’s gut, and still represents, on average, 1% of the total operational taxonomic unit at 1 year. For 24% of the virome samples, presence of coliphages could be seen as indicator strains were identified. A collection of 75 isolated bacteriophages was further tested for its host range over a selection of 75 *E. coli* strains representative of the cohort. Twenty-eight percent of them were virulent and the remaining ones temperate. Strain response was markedly different towards the bacteriophage life cycle: whereas 93% of the tested bacterial strains were susceptible to at least one virulent bacteriophage, only 18% were sensitive to at least one temperate bacteriophage. 30% of the virulent bacteriophages were shown to kill at least 30% of the strains, whereas none of the temperate bacteriophages could kill 30% of the strains. In the light of phageome sequence analyses, the experimental results suggested that all bacteriophages differ in their impact on the microbiota.

**Camille d’Humieres** (graduate student in the group of Edouardo Rocha, UMR 1137 and UMR3525, Paris) studies the impact of antibiotics on the human gut microbiome [8] and phageome by monitoring bacteriophage dynamics before, during and after treatments. They took advantage of a prospective, single-centre, open, randomized clinical trial involving 22 healthy volunteers treated by the intravenous route with either ceftriaxone (1 g/24 h) or cefotaxime (1 g/8 h) for three days. C. d’Humieres compared several experimental methods to concentrate and purify viral particles, and was able to retain the PEG-concentration, which allowed him to follow 22 sets of contigs over time. Preliminary results show that diversity differs in the presence of each cephalosporin. Perturbations of the gut phageome were stronger when treated by ceftriaxone than cefotaxime. Moreover, when treated with ceftriaxone, the predominant bacteriophage contigs showed a drastic decrease just after treatment, and a resilience of the community ten days after treatment. 

Moving more towards the field of computational analysis of bacteriophages, **Ariane Bize**, (Irstea, UR HBAN, Antony) presented data about k-mer approaches to study bacterial genomes and their mobile elements. K-mer approaches, which are annotation independent, have greatly developed in recent years, largely driven by the advent of next-generation sequencing. Their speed automation and accuracy are major advantages. The interest of applying k-mer approaches to study the mobilome is only starting to be explored, with many efforts put towards viral metagenomics. Ariane Bize evaluated the potential of applying simple k-mer approaches to understand the evolutionary history of mobile elements by focusing on viruses and plasmids from the domain *Archaea*. A. Brize selected more than 590 cell, virus and plasmid genomes, originating from 11 distinct orders of archaeal cells, and subsequently implemented multivariate and statistical analyses to identify the factors underlying the 5-mer composition of mobile element genomes in the domain *Archaea*. Genomes overall grouped according to the host order, except for the Haloarchaea. Within each group, cells tended to cluster together, while viruses and plasmids tended to cluster according to their own taxonomic family. The observed pattern likely results from the combined influence of co-evolution and environmental constraints. It confirms the potential of the k-mer signal for extrachromosomal contig analysis, in particular for their taxonomic assignation. Another application could be the detection of singular evolutionary trajectories by focusing on outliers. Indeed, previously-known and one novel case of recent HGT were efficiently detected during the present study. 

## 3. Bacteriophage-Host Molecular Interaction

This session started with a keynote lecture from **Jennifer Mahony** (University College Cork, Ireland) entitled “Some like it sweet – host recognition by bacteriophages of lactic acid bacteria”. This refers to the nature of the receptors of these bacteriophages at the surface of their Gram positive host, which are sugars. Lactococcal bacteriophages have been the focus of intense research due to the economic loss they can induce in the dairy fermentation industry. Lactococcal bacteriophages have an “exquisitely” narrow host range, borne by receptor binding proteins that share structural folds and sequence similarity with the exception of the receptor binding domain. This latter domain can be swapped between bacteriophages, thereby swapping host specificity. What about the polysaccharides at the surface of the different *Lactococcus* strains? There are three defined genotype groups depending on the cell-wall encoding polysaccharide operons and with further groups expected based on PCR typing data. The diversity comes from the genetic diversity of the glycosyltransferases and presumably encodes a variety of sugars arranged in penta- and hexa-saccharides on the cell surface. These sugars are a signature of the particular strain and explain the high degree of host specificity of lactococcal bacteriophages.

The next presentation by **Lia Marques Godinho** (post-doc in the group of Paulo Tavares, I2BC UMR 9198, Gif-sur-Yvette) provided evidence about the importance of the chaperone GroEL in the biogenesis of bacteriophages. Molecular chaperones play an essential role in the folding of nascent chain polypeptides, refolding of misfolded proteins, and other house-keeping and stress-related functions. SPP1 is a dsDNA, lytic Siphophage infecting the Gram positive *Bacillus subtilis*. During its infection cycle, SPP1 massively hijacks the host resources, leading to the synthesis of >9 Mbp of viral DNA and 150,000 polypeptide chains to produce 200 virions in 30 min, highly challenging the cell. The goal of this work is to investigate the dependence of SPP1 on *B. subtilis* GroEL. To this purpose, L. Marques Godinho inactivated *groEL* by knock out and investigated the infection dynamics of SPP1, SPP1 DNA replication and viral particle assembly on the mutant strain. The most affected step of the SPP1 infection was the formation of viral particles, and more specifically bacteriophage capsid assembly. A similar role of GroEL in the morphogenesis of *Enterobacteriaceae* bacteriophages was shown.

**Mathieu de Jode** (graduate student in the group of Laurent Debarbieux, Institut Pasteur, Paris) discussed how a single bacteriophage protein efficiently disrupts the host physiology by targeting sigma factors. A bacterial cell infected by a bacteriophage becomes a viral factory (or virocell) as most of its resources are dedicated to virion production [6]. To investigate this transformation of the bacteria into a virocell, the group is studying the infection of the opportunistic pathogen *Pseudomonas aeruginosa* by bacteriophage PAK_P3. Using a global transcriptomic approach, M. de Jode found that PAK_P3 alters the transcription of over a thousand host genes and temporally regulates the expression of its own genes [9]. Amongst the early expressed bacteriophage genes, the scientists focused on gp92, which is one of the few non-structural genes conserved between Kpp10virus and Pakpunavirus genera [10]. 

Finally, **Isabelle Bertrand** (MC U. Lorraine, LCPME UMR7564, Nancy) reported on the efficacy of different treatments to inactivate enteric viruses. Heat and chlorine treatments were considered as they are the most frequently used treatments to inactivate viruses in the food industry and water treatment, respectively. The effects of heat and chlorine on the physico-chemical characteristics of viral particles are still poorly understood. I. Bertrand presented data on the MS2 F-specific F-RNA bacteriophage that is used as a model for pathogenic enteric viruses. Using a concentrated bacteriophage lysate (10^14^ UFP/mL), she showed that exposure to heat should exceed 72 °C and 10 min to massively inactivate and provoke physical disruption of the bacteriophage capsids. In contrast, exposure to 200 mg/mL hypochlorite for 10 min was not sufficient to damage the bacteriophages seriously. A combination of both moderate heat and hypochlorite however resulted in an optimal effect. The observed appearance of hydrophobic domains on the surface of capsids during treatment of viruses could play an important role in distinguishing between infectious and inactivated viruses.

## 4. Therapy and Biotechnology Applications

The third session was dedicated to therapy and framework regulation issues, as well as other biotechnology applications of bacteriophages. It opened with the key lecture of **Joana Azeredo** (Centre of Biological Engineering University of Minho, Portugal) entitled “bacteriophages/biofilm interaction: strategies to improve bacteriophage efficacy against infectious biofilms”. About 80% of the microbial biomass is in a biofilm form, which forms bacterial reservoirs. This has major clinical implications (chronic wounds, otitis media, osteomyelitis, lung infection, contamination of materials including lenses, heart valves, catheters and orthopedic prostheses) and novel treatments of contaminated abiotic surfaces are urgently needed. Since 2005, different research groups have studied the killing activity of bacteriophages on hosts established as biofilms (*Pseudomonas fluorescens*, *Staphylococcus epidermidis*, *Acinetobacter spp*). The expertise of the Minho’s group allowed the identification of three major challenges faced by bacteriophages when interacting with sessile bacteria. First, the nature of the matrix reduces the efficiency of the treatment. Through an elegant study based on FISH, the bacteriophage phiBB-PAA2 was shown to use water channels to penetrate the biofilm structure and target its host, *P. aeruginosa*. Efficiency of the treatment was increased through mechanical debridement of the biofilm. Hence, the logarithmic reduction achieved for *S. epidermis* in the presence of PhiIBB-sep1 was increased from 0.5 (control) to 3 when mechanical disruption of the biofilm was carried out prior to bacteriophage treatment. Another parameter to address is the heterogeneity of the cells entrapped in the matrix with regard to their physiology (active and dormant cells). Last, the presence of persistent cells in the population as well as the emergence and rapid proliferation of resistant cells in the biofilm upon bacteriophage treatment are of great concern. Successful reduction of biofilms using lytic bacteriophages should consider (a) a careful selection of bacteriophages able to infect dormant cells, (b) a mechanical/enzymatic disruption of the matrix, and (c) the combination of cocktail of bacteriophages or combined therapies with antibiotics.

**Martine Boccara** (ESPCI, Paris). Nitrogen may well be the most studied nutrient in agricultural systems. The application of fertilizer in excessive amounts is known to pollute rivers [11] and impact water quality. To overcome this problem, denitrification is currently used in water purification. It is a microbially facilitated process where nitrate is reduced and ultimately produces molecular nitrogen. Despite favorable physicochemical conditions, denitrification is not performed at maximum yield. M. Boccara hypothesizes that bacteriophages could influence denitrification in sewage treatment plants or rivers. Bacteriophages infecting denitrifying bacteria were successfully isolated from a river, and were shown to act as a lysogen. Using interferometric microscopy, bacteriophage particles were observed in mitomycin-induced culture supernatants. Their density and heterogeneity in size suggest that they may correspond to membrane vesicles. Such entities have been shown to be the vehicles of protein, nucleic acids between bacteria and more recently for viruses [12]. 

**Fernando Clavijo** (graduate student, within the groups of Mireille Ansaldi, Laboratoire de Chimie Bactérienne UMR 7283, Marseille and Marie-Agnès Jacques, IRHS UMR 1345, Angers) investigates the use of bacteriophages to cure plant infections, and focuses on *Xylella fastidiosa* (*Xf*). The pathogen is a slow growing Gram-negative plant-pathogenic bacterium emerging in Asia and Europe, is transmitted by xylem feeding insects and belongs to the *Xanthomonadaceae* family. A few bacteriophage cocktails recently became commercially available to treat diseases caused by *Xanthomonadaceae* or *Ralstonia solanacearum* [13]. However, they do not specifically target *Xf*. In his presentation, F. Clavijo reported the isolation and preliminary characterization of various lytic bacteriophages (Podo- and Siphoviridae) isolated from different environmental sources. 

In order to answer the question of the posology to be used in bacteriophage therapy, **Raphaëlle Delattre** (medical doctor and graduate student in the group of Laurent Debarbieux, Institut Pasteur, Paris and Jean-Damien Ricard, Hôpital Louis Mourier, Service de Réanimation Médico-Chirurgicale, Colombes) investigated the pharmacokinetics (concentration-time courses in the body resulting from administration of a drug dose) and pharmacodynamics (observed effects resulting from a given drug concentration) of several bacteriophages targeting *E. coli* in mice. First, R. Delattre recorded *E. coli* bacteriophage distributions over time into several organs (lungs, spleen, liver, kidneys) and blood compartments. Following nasal administration, bacteriophages were not eliminated rapidly and remained infectious, with a low systemic passage from lung to blood. When applied by intravenous injection, bacteriophage half-life was short (a few hours) and their rapid translocation in the lung was observed. Second, they presented preliminary data on the biodistribution over time in the organs and blood compartments of bacteriophages targeting *P. aeruginosa* administrated by intranasal or systemic routes. 

**Frédéric-Antoine Dauchy** (medical doctor, Centre Hospitalier Universitaire “Pellegrin”, Bordeaux) presented PHAGOS, a multicentric, open randomized clinical trial, whose management and coordination is ensured by the Bordeaux Hospital. Scheduled to start in 2019, PHAGOS is a phase I/II study of tolerance and efficacy of bacteriophage therapy added to standard surgical treatment and antibiotics in adults with relapsing staphylococcal prosthetic joint infections of the hip and knee.

This session ended with a lecture by **Gilbert Verbeken** (Hopital Militaire de la Reine Astrid, Brussels, Belgium) who presented the legal framework that may be applicable to bacteriophage therapy in Western medicine. G. Verbeken first provided the historical background of the use of bacteriophage therapy to treat military injuries in Finland (during World war II), Germany (in North Africa during World war II), United Kingdom (in India), Japan and USSR. Recent decades have seen a decline in bacteriophage therapy in Western countries, with marginal use in Poland, although bacteriophage therapy and commercial production continued in Georgia and Russia. After presenting several case reports [14,15], the use of bacteriophage therapy on a large scale [16] and the difficulties in setting up clinical trials [17], G. Verbeken moved to the future of bacteriophage therapy as no bacteriophage-based treatment is so far commercialised in the European Union. He began thinking about the future development of bacteriophage therapy under the current legal framework for medicines for human use (“Conventional Medicinal Product licensing pathways”) adopted by the European commission on the 29 of March 2011. This status requires the following elements: (i) the manufacturing of bacteriophages according to Good Manufacturing Procedures (GMP), (ii) the setting of preclinical studies and (iii) phase I, II and III clinical trials, and (iv) marketing. The current framework may be inappropriate and could expand the time for development of bacteriophage therapy, as studies will be time-consuming (an average of 10 years is required to get an antibiotic to market) and expensive (1 million euros per product). G. Verbecken recalled that these medicinal product licensing pathways were developed for conventional drugs such as antibiotics that are chemical molecules to be broadly sold in pharmacies. This is not the case for bacteriophages, which are natural biological entities already present in our body. G. Verbeken also questioned the rationale of authorizing fecal transplantation [18] that contains billions of unknown bacteriophages, while bacteriophage therapy is not permitted even though it uses well defined and characterized entities. Based on these arguments, the Belgium national parliament on 26 October 2016 took the decision to assign bacteriophages under the legislation of “active pharmaceutical ingredient” after the publication of a monography defining the rules of characterization and production of bacteriophage preparations [19]. This has introduced more flexibility allowing the military hospital to treat an increasing number of patients [20]. The presentation finished with a list of further investigations that are needed on the scientific side, but also on a call to politicians to change European regulations. 

Finally, we had the opportunity to listen to **Pilar Garcia** who presented the different groups working in Spain on bacteriophages that are part of a network founded several years ago “Spanish Network of Bacteriophages and Transducter Elements (FAGOMA)”. 

Overall, the annual meeting of the French scientific community working on the interaction between bacteriophages and their hosts has been a rich and very stimulating symposium, illustrated by the diversities of topics addressed, and the development of high-throughput technologies and tools. An important observation was the increasing number of model systems to study bacteriophage–host interactions, raising exciting ecological, agricultural and medical perspectives. The French network acknowledges the presence of medical doctors, and will continue its efforts to encourage clinicians interested in bacteriophage therapy to join our network and meetings (either annual conference or workshops). Our 2019 conference is scheduled on October 8–9, at the Institute for Structural Biology in Grenoble. 

## Figures and Tables

**Table 1:** Titles of the posters presented during the 4th annual conference of the French bacteriophage network (bacteriophages.fr) at the University Bordeaux in 2018.

Poster Title	Presenter (Group)
Bacteriophages gouvern dynamic of diversity of bacterial resistance.	Sylvain Gandon, Hélène Chabas, Enrique Ortega Abboud & Antoine Nicot
Prophages and other mobile genetic elements as regulatory switches.	Fanny Wegner & Eduardo Rocha.
Nitric oxide-driven prophage maintenance involves unsuspected activity of NorV(W) reductase.	Stéphanie Champ, Maëlle Delannoy, Alice Boulanger, Yann Denis, Gaël Brasseur & Mireille Ansaldi
AppY, a bacteriophage-encoded protein central to the bacterial regulatory network.	Naoual Derdouri, Nicolas Ginet, Mireille Ansaldi & Aurélia Battesti
Analysis of adhesin in T4 bacteriophages and search for a synergistic effect between lytic bacteriophages and antibacterial bio-molecules.	Sabrina N. Trojet, Bouchra El Khalfi & Abdelaziz Soukri
Characterization of the long elusive nuclease of bacteriophage T5.	Ombeline Rossier, Léo Zangelmi, Madalena Renouard & Pascale Boulanger
Study of the structure of T5 bacteriophage tail tip complex complex using cryo-electron microscopy.	Romain Linares, Charles Arnaud, Gregory Effantin, Guy Schoehn & Cécile Breyton
Walking through the dynamic bacteriophage-bacteria interactions in the digestive tract using DNA contacts.	Quentin Lamy-Besnier, Marta Lourenço, Martial Marbouty, Louisa de Sordi, Romain Koszul & Laurent Debarbieux
Viruses and membrane vesicles produced by denitrifying bacteria.	Roose-Amsaleg C., Yasmina Fedala, Venien-Bryan C. & Martine Boccara
Characterization of new bacteriophages for the biocontrol of a plant bacterial pathogen.	Lisa Le Joncour, Stéphane Poussier & Clara Torres-Barceló
New optical method for detecting and counting biotic nanoparticles.	Yasmina Fedala, Martine Boccara & Claude Boccara
In vitro and in vivo resistance to the therapeutic bacteriophage 536_P1.	Baptiste Gaborieau, Raphaëlle Delattre, Laurent Debarbieux & Jean-Damien Ricard
Isolation and characterization of bacteriophages targeting *Flavobacterium psychrophilum*, a pathogenic bacterium in farmed trout.	Thomas Guiraud, Cécile Philippe, Fety Jaomanjaka, Etienne Gontier, Claire Le Marrec & Michel Le Hénaff
Addressing knowledge gaps in the taxonomy of bacteriophages of lytic acid bacteria: bacteriophage-host interactions in the underrepresented genera *Oenococcus, weisella* and non-dairy *Leuconostoc* spp.	Claire Le Marrec, Olivier Claisse, Fety Jaomanjaka, Cécile Philippe, Amel Chaib, Coralie Coudray-Meunier, Sarah Chuzeville, Valérie Gabriel & Cathy Faucher
Survey on prophages infecting lactic acid bacteria *Weisella cibaria* and *W. confusa.*	Coralie Coudray-Meunier, Valérie Gabriel, Claire Le Marrec & Catherine Fontagné-Faucher
Oenological environment as a source of a novel Tectivirus.	Cécile Philippe, Mart Krupovic, Fety Jaomanjaka, Olivier Claisse, Melina Petrel & Claire Le Marrec

## References

[1] Luria S.E., Delbrück M. (1943). Mutations of Bacteria from Virus Sensitivity to Virus Resistance. Genetics.

[2] Hershey A.D., Chase M. (1952). Independent Functions of Viral Protein and Nucleic Acid in Growth of Bacteriophage. J. Gen. Physiol..

[3] Roberts R.J. (2005). How restriction enzymes became the workhorses of molecular biology. Proc. Natl. Acad. Sci. USA.

[4] Barrangou R., Fremaux C., Deveau H., Richards M., Boyaval P., Moineau S., Romero D.A., Horvath P. (2007). CRISPR Provides Acquired Resistance Against Viruses in Prokaryotes. Science.

[5] Pirnay J.-P., Verbeken G., Rose T., Jennes S., Zizi M., Huys I., Lavigne R., Merabishvili M., Vaneechoutte M., Buckling A. (2012). Introducing yesterday’s phage therapy in today’s medicine. Future Virol..

[6] Forterre P. (2011). Manipulation of cellular syntheses and the nature of viruses: The virocell concept. C. R. Chim..

[7] Keesing F., Ostfeld R.S. (2015). Is biodiversity good for your health?. Science.

[8] Burdet C., Grall N., Linard M., Bridier-Nahmias A., Benhayoun M., Bourabha K., Magnan M., Clermont O., d’Humières C., Tenaillon O. (2019). Ceftriaxone and cefotaxime have similar effects on the intestinal microbiota in human volunteers treated by standard doses regimens. Antimicrob. Agents Chemother..

[9] Chevallereau A., Blasdel B.G., Smet J.D., Monot M., Zimmermann M., Kogadeeva M., Sauer U., Jorth P., Whiteley M., Debarbieux L. (2016). Next-Generation “-omics” Approaches Reveal a Massive Alteration of Host RNA Metabolism during Bacteriophage Infection of Pseudomonas aeruginosa. PLoS Genet..

[10] Henry M., Bobay L.-M., Chevallereau A., Saussereau E., Ceyssens P.-J., Debarbieux L. (2015). The Search for Therapeutic Bacteriophages Uncovers One New Subfamily and Two New Genera of Pseudomonas-Infecting Myoviridae. PLoS ONE.

[11] Xia X., Zhang S., Li S., Zhang L., Wang G., Zhang L., Wang J., Li Z. (2018). The cycle of nitrogen in river systems: Sources, transformation, and flux. Environ. Sci.: Processes Impacts.

[12] Tzipilevich E., Habusha M., Ben-Yehuda S. (2017). Acquisition of Phage Sensitivity by Bacteria through Exchange of Phage Receptors. Cell.

[13] Buttimer C., McAuliffe O., Ross R.P., Hill C., O’Mahony J., Coffey A. (2017). Bacteriophages and Bacterial Plant Diseases. Front. Microbiol..

[14] Duplessis C., Biswas B., Hanisch B., Perkins M., Henry M., Quinones J., Wolfe D., Estrella L., Hamilton T. (2018). Refractory Pseudomonas Bacteremia in a 2-Year-Old Sterilized by Bacteriophage Therapy. J. Pediatric Infect. Dis. Soc..

[15] Jennes S., Merabishvili M., Soentjens P., Pang K.W., Rose T., Keersebilck E., Soete O., François P.-M., Teodorescu S., Verween G. (2017). Use of bacteriophages in the treatment of colistin-only-sensitive Pseudomonas aeruginosa septicaemia in a patient with acute kidney injury—a case report. Crit. Care.

[16] Babalova E.G., Katsitadze K.T., Sakvarelidze L.A., Imnaishvili N.S., Sharashidze T.G., Badashvili V.A., Kiknadze G.P., Meĭpariani A.N., Gendzekhadze N.D., Machavariani E.V. (1968). Preventive value of dried dysentery bacteriophage. Zh. Mikrobiol. Epidemiol. Immunobiol..

[17] Jault P., Leclerc T., Jennes S., Pirnay J.P., Que Y.-A., Resch G., Rousseau A.F., Ravat F., Carsin H., Le Floch R. (2019). Efficacy and tolerability of a cocktail of bacteriophages to treat burn wounds infected by Pseudomonas aeruginosa (PhagoBurn): A randomised, controlled, double-blind phase 1/2 trial. Lancet Infect. Dis..

[18] Van Nood E., Vrieze A., Nieuwdorp M., Fuentes S., Zoetendal E.G., de Vos W.M., Visser C.E., Kuijper E.J., Bartelsman J.F.W.M., Tijssen J.G.P. (2013). Duodenal Infusion of Donor Feces for Recurrent Clostridium difficile. N. Engl. J. Med..

[19] Pirnay J.-P., Verbeken G., Ceyssens P.-J., Huys I., De Vos D., Ameloot C., Fauconnier A. (2018). The Magistral Phage. Viruses.

[20] Djebara S., Maussen C., de vos D., Merabishvili M., Damanet B., Pang K.W., de Leenheer P., Strachinaru I., Soentjens P., Pirnay J.-P. (2019). Processing Phage Therapy Requests in a Brussels Military Hospital: Lessons Identified. Viruses.

